# Demographics, clinical interests, and ophthalmology skills confidence of medical student volunteers and non-volunteers in an extracurricular community vision screening service-learning program

**DOI:** 10.1186/s12909-022-03194-0

**Published:** 2022-03-04

**Authors:** Eleanor Burton, Lama Assi, Hursuong Vongsachang, Bonnielin K. Swenor, Divya Srikumaran, Fasika A. Woreta, Thomas V. Johnson

**Affiliations:** 1 Johns Hopkins University School of Medicine, Baltimore, MD USA; 2 Wilmer Eye Institute, Johns Hopkins University School of Medicine, 600 N Wolfe Street, Maumenee B-110, Baltimore, MD 21287 USA

**Keywords:** Graduate Medical Education, Health Disparity, Minority and Vulnerable Populations, Vision Screening, Ophthalmology

## Abstract

**Background:**

Medical school curricular hours dedicated to ophthalmology are low and declining. Extracurricular ophthalmology activities, such as participation in community vision screenings, may serve an important adjunctive role in medical school curricula. The Johns Hopkins University (JHU) **V**ision **S**creening **I**n **O**ur **N**eighborhoods (ViSION) Program is an example of a voluntary medical student-directed community service-learning program.

**Methods:**

We used a mixed-methods cross-sectional approach, including an online survey and semi-structured interviews. JHU School of Medicine students enrolled in MD or MD/PhD programs during the 2019–2020 academic year were surveyed regarding demographics, career and service interests, involvement in ophthalmology-related activities, and confidence in their ophthalmology-related skills. Survey responses were compared between ViSION volunteers and non-volunteers using Fisher’s exact chi-square tests. Semi-structured interviews were conducted via webconference with 8 prior or current ViSION volunteers and responses analyzed using inductive thematic analysis. Data were collected when ViSION volunteers were in variable stages of their medical education and involvement with the ViSION program.

**Results:**

A total of 118 medical students were included, representing an overall response rate of 24.6% of JHU medical students. ViSION volunteers reported greater involvement in ophthalmology-related research (42% vs. 4%, *p* < 0.001), intent to apply to ophthalmology residency programs (35% vs. 1%, *p* = 0.001), and confidence with multiple ophthalmology knowledge and clinical skill domains. In particular, ViSION volunteers were more likely to feel confident estimating cup-to-disc ratio using direct ophthalmoscopy (20% vs. 0%, *p* < 0.001). In open-ended survey and interview questions, most volunteers attributed at least some degree of their ophthalmology skill development and desire to pursue ophthalmology and public health careers to their ViSION experience.

**Conclusions:**

Medical students who volunteered with a student-led community vision screening program were more likely to have a prior interest in ophthalmology than those who did not volunteer, but only 1/3 of volunteers planned to pursue a career in ophthalmology. Overall, volunteers reported higher confidence performing ophthalmology-related clinical skills, suggesting that student-led community vision screening programs may provide an important avenue for medical students to explore public health aspects of ophthalmology, while practicing ophthalmology exam skills and learning about common ophthalmic pathologies, regardless of their career intentions.

**Supplementary Information:**

The online version contains supplementary material available at 10.1186/s12909-022-03194-0.

## Background

In 2017, an estimated 93 million people were at high risk for vision loss in the United States [1]. Diagnosing and managing eye disease is essential to preventing or slowing the progression of vision loss. However, multiple assessments of basic ophthalmology clinical skills in medical students and non-ophthalmologist physicians highlight a lack of adequate proficiency [2–5]. At the same time, there has been a progressive decline in ophthalmology curricular hours during medical school education. In 2018, only 16% of the medical schools affiliated with the Association of University Professors of Ophthalmology (AUPO) required a clinical clerkship in ophthalmology [6].

Nontraditional teaching opportunities in ophthalmology may be useful for filling important gaps in medical education. During the COVID-19 pandemic, several medical schools developed virtual ophthalmology learning experiences for medical students [5], a model that has been demonstrated to improve medical student performance in ophthalmology [7]. Among AUPO-affiliated medical schools, 75% provide community service experiences in ophthalmology [6]. A number of these initiatives have been described in the literature [8–10], but data regarding their impact on medical education is scarce [9, 11, 12]. Outside ophthalmology, participation in medical service-learning activities has been shown to increase students’ empathy, comfort interacting with patients, and knowledge about disease prevention [13–15]. Moreover, service activities provide students with opportunities to work in interprofessional teams, gain peer-to-peer teaching experience, be more civically engaged [13, 14, 16–18], and gain exposure to primary care and social justice [19, 20]. Within ophthalmology, studies have shown that self-directed learning, peer-assisted learning, and problem-based learning are useful strategies for optimizing medical student education [21–23]. However, it is unclear how medical service-learning opportunities in ophthalmology specifically benefit students. Ophthalmology community service opportunities are relevant to those who are already interested in ophthalmology and may be looking for an avenue to improve their ophthalmology knowledge and skills, or to further explore their research and career interests. These opportunities may also be valuable for students without a particular interest in ophthalmology, but who are interested in community outreach and helping underserved populations in general.

To gain insight into how service-learning opportunities in ophthalmology might augment medical school education, we examined student involvement with the Johns Hopkins University’s Vision Screening In Our Neighborhoods (JHU ViSION) Program (formerly called the JHU Student Sight Savers Program). This organization is run primarily by JHU School of Medicine (JHU-SOM) medical students with mentorship from faculty ophthalmologists of the Wilmer Eye Institute. During the academic calendar year, ViSION holds one 4-h-long screening event every 1–2 months. Volunteers operate 6 different stations at each event: check-in and check-out, visual acuity testing, visual field assessment using automated perimetry, intraocular pressure using an ocular rebound tonometer, cup-to-disc ratio assessment using direct ophthalmoscopy, and imaging of the macula and optic nerve head using optical coherence tomography (OCT). Prior to volunteering, students participate in a 90-min didactic session with ophthalmology faculty and undergo at least 2 h of hands-on training using the equipment. Medical students interested in using the direct ophthalmoscope or OCT require additional training sessions. Thus, even volunteers who opt for the minimum level of training and attend only 1 screening event spend 6 h actively immersed in ophthalmology. This is significant given that the median medical school curricula includes only 7 h of pre-clinical ophthalmology training [6]. Following completion of this training, students engaged in ViSION partner with local community organizations to conduct vision screenings for residents of Baltimore City, under direct supervision by faculty ophthalmologists. Screened individuals needing follow-up care are evaluated and treated by the volunteer faculty ophthalmologists at the Wilmer Eye Institute regardless of their insurance status or ability to pay.

## Methods

The purpose of this study is to describe the demographics, interests, and self-confidence in ophthalmology-related knowledge and clinical skills of JHU-SOM medical students who volunteered with ViSION versus those who did not. In addition, we aimed to explore a small subset of volunteers’ perceptions regarding the extent to which their volunteer experiences influenced their interests and clinical confidence.

We used a sequential, mixed-methods approach. First, we conducted a descriptive survey among JHU-SOM students, comparing ViSION volunteers with non-volunteers. Subsequently, we conducted one-on-one, semi-structured, in-depth interviews with a small number of ViSION volunteers to expand upon our quantitative findings by elucidating the role of ViSION in these students’ medical education. A raffle for one of four $50 Amazon gift cards was offered for participation in the study.

### Ethical approval and consent to participate

This quality improvement and assessment study, including written survey and semi-structured interviews, was reviewed and acknowledged as exempt by the Johns Hopkins Medicine Institutional Review Board (IRB00233696). The research methods used in this study adhere to the Declaration of Helsinki. Completion of the survey included provision of informed consent to participate. Interview participants provided consent to participate and for publication of their responses.

### Quantitative data collection and analysis

All enrolled JHU-SOM medical students were invited to participate in the online survey via email in the Winter and Spring periods during the 2019–20 academic year. Survey responses were collected for a period of 1 month following the initial request, with reminder emails sent at 2 and 3 weeks after the initial request. Students actively enrolled in the MD or MD/PhD programs who completed the questionnaire were included in the analyses.

This 45-question online survey was designed to describe student demographics, involvement with ViSION, confidence in ophthalmology knowledge and skills, interest in ophthalmology-related activities, and involvement and interest in working with underserved populations (Additional File 1). In particular, survey respondents were asked to rate their confidence performing the following ophthalmology-related clinical skills on a 5-point Likert scale: 1) managing, triaging, or understanding ophthalmology problems encountered in a clinical setting, 2) describing and explaining eye problems and treatments to patients, 3) assessing a patient’s visual acuity, 4) assessing a patient’s peripheral visual field, 5) measuring a patient’s intraocular pressure, 6) estimating a patient’s cup-to-disc ratio with fundoscopy, and 7) evaluating a patient’s retina using an OCT. Survey respondents were also asked to rate how confident they felt in their understanding of ophthalmology-related content as encountered in the following settings: 1) 1^st^ and 2^nd^ year of medical school curricular exams, 2) clinical shelf exams, and 3) USMLE step exams. All respondents were also asked to estimate their level of interest and engagement with ophthalmology-related material during medical school. ViSION volunteers were directed to an additional set of questions that asked about the duration (in years) of their involvement with ViSION, the number of screening events they had attended, the position(s) that they held at screening events, and whether they were part of the ViSION leadership board. In addition, ViSION volunteers were asked to describe (on a 5-point Likert scale) the degree to which they attributed their responses to previous interest- and confidence-related questions to their involvement with ViSION. Survey participants who indicated involvement with ViSION were also asked to respond to several free-form questions regarding their motive for joining, continuing, and terminating involvement with ViSION if applicable. The survey was administered using Qualtrics^XM^ software (Qualtrics, Provo, UT). Stata Software, version 15 (StataCorp, College Station, TX) was used for data analysis. Responses between ViSION and non-ViSION medical students were descriptively analyzed and compared with Fisher’s exact chi-square test. The significance level was set at *p* < 0.05. Survey responses were well-aligned with the expected results and internally correlated within thematic groups, supporting the validity of the survey.

### Qualitative data collection and analysis

A key informant strategy was used for interview recruitment, where pre-clinical and clinical medical students who had the greatest involvement with ViSION based on number of screenings attended were invited via email in December 2020 to participate in one-on-one, semi-structured interviews. Students were recruited until thematic saturation was achieved. Interviews were conducted via webconference (Zoom Video Communications, Inc., Version 5.4.7) in January 2021.

An interview guide with open-ended questions was developed based on the survey results and answers to the free-form questions. The questions aimed to explore students’ motives behind volunteering with ViSION, and the role that participation played in shaping their interests, ophthalmology knowledge and skills, and career plans (Additional File 2). Study authors HV and LA conducted all one-on-one interviews; HV interviewed the participants, while LA facilitated and took notes. Interviews lasted 30 min, were audio-recorded, then transcribed verbatim. A codebook was developed (Additional File 3) using an iterative approach based on the first 2 interviews. Following development of the codebook, all transcripts were then coded on NVivo (QSR International, Version 12) and themes generated using inductive thematic analysis [24]. Interrater reliability was established at κ = 0.78. ViSION volunteer responses were consistent across survey and interview questions, supporting the reliability of our survey.

## Results

### Quantitative survey

A total of 118 medical students from the JHU-SOM were included, representing a response rate of 24.6% of JHU-SOM medical students (Supplemental Figure S1). Participants ranged in age from 23–35 years with an average ± standard deviation age of 26.4 ± 2.8 years, the majority were female (61.9%), and most participants were White (56.8%) or Asian (32.2%). Twenty-six (22%) participants were current for former ViSION volunteers, most of whom participated in the program for 1–2 years (53.8%) and attended a median of 2 screening events (Table 1). ViSION participants and non-participants had similar distributions of demographic characteristics. For comparison, the overall population of students enrolled in the MD program at JHU-SOM in 2020–21 ranged in age from 20–37 years with an average age of 26 years, 51% were female, 38% White, and 16% characterized as “underrepresented in medicine,” a term attributed to all racial and ethnic populations that are underrepresented in medical professions relative to their proportions in the general population.

**Table 1:** Characteristics of survey respondents

		Overall	ViSION Volunteers	Non-ViSION Volunteers	*P*-value^a^
Sample Size, N (%)		118 (100%)	26 (100%)	92 (100%)	
Gender, N (%)	*Female*	73 (61.9%)	14 (53.8%)	59 (64.1%)	
	*Male*	44 (37.3%)	12 (46.2%)	32 (34.8%)	
	*Other*	1 (0.8%)	0 (0%)	1 (1.1%)	0.502
Race, N (%)	*Asian*	38 (32.2%)	12 (46.2%)	26 (28.3%)	
	*Black or African American*	6 (5.1%)	2 (7.7%)	4 (4.3%)	
	*Multiple/Other*	7 (5.9%)	1 (3.8%)	6 (6.5%)	
	*White*	67 (56.8%)	11 (42.3%)	56 (60.9%)	0.211
Age, mean ± SD	*Range: 23–35*	26.4 ± 2.8	25.8 ± 2.3	26.5 ± 2.9	0.721
Medical training year, N (%)	*MS1*	35 (29.7%)	7 (26.9%)	28 (30.4%)	
	*MS2*	26 (22%)	4 (15.4%)	22 (23.9%)	
	*MS3*	20 (16.9%)	7 (26.9%)	13 (14.1%)	
	*MS4*	37 (31.4%)	8 (30.8%)	29 (31.5%)	0.463
Duration of ViSION Involvement, N (%)	< *1 year*	-	9 (34.6%)	-	
	*1–2 years*	-	14 (53.8%)	-	
	*2–3 years*	-	2 (7.7%)	-	
	> *5 years*	-	1 (3.8%)	-	
Number of Screenings Attended, median (IQR)	*Range: 0–25*		2 (1, 5)		

Abbreviations: *N* number of respondents, IQR: interquartile range;

^a^P-values computed using Fisher's exact chi-square test comparing ViSION volunteers to non-volunteers

**Fig. 1:**
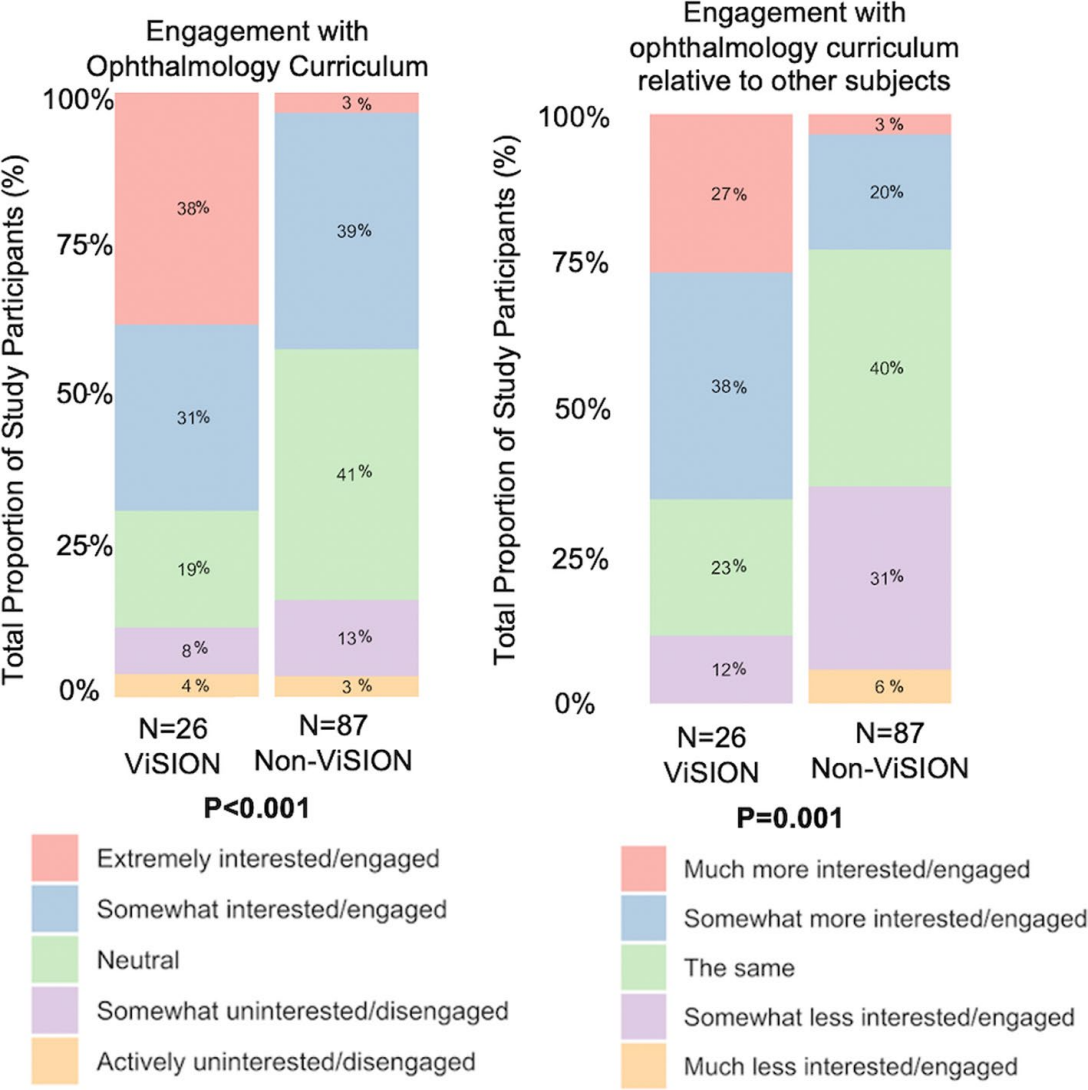
Survey participant engagement with the ophthalmology curriculum. Stacked bar graphs depict the percentage of respondents that answered each question as indicated in the color-coded legend. Respondents are divided according to whether or not they had volunteered in the JHU-SOM ViSION Program. P-values indicate results of statistical testing using Fisher’s exact Chi-square test

A greater fraction of ViSION volunteers indicated an interest in pursuing a career in ophthalmology upon starting medical school (45.8% vs. 6.7%, *p* < 0.001) compared with non-volunteers (Table 2). Additionally, a higher proportion of ViSION volunteers reported involvement in ophthalmology research (42.3% vs. 4.3%, *p* < 0.001) and clinical ophthalmology electives (23.1% vs. 0%, *p* < 0.001), and a larger proportion applied to or planned to apply to an ophthalmology residency (34.6% vs. 1.1%, *p* < 0.001, Table 2). ViSION volunteers reported greater interest and engagement with ophthalmology-related content encountered in the medical school curriculum (69% somewhat or extremely interested/engaged vs. 42%, *p* < 0.001, Fig. 1). When comparing their interest and engagement with the ophthalmology curriculum relative to other subjects, 65% of ViSION volunteers were somewhat or much more interested or engaged, whereas only 23% of non-ViSION volunteers reported the same (*p* = 0.001, Fig. 1).

**Table 2:** Participant interests

		Overall	ViSION Volunteers	Non-ViSION Volunteers	P-value^a^
Particular interest in ophthalmology at med school entry, N (%)	*Yes*	17 (15%)	11 (45.8%)	6 (6.7%)	< 0.001
Particular interest in ophthalmology currently, N (%)	*Yes*	10 (8.5%)	9 (34.6%)	1 (1.1%)	
	*Unsure*	9 (7.6%)	5 (19.2%)	4 (4.3%)	< 0.001
Ophthalmology Interest Group participant, N (%)	*Yes*	17 (14.4%)	13 (50%)	4 (4.3%)	< 0.001
Ophthalmology research involvement, N (%)	*Yes*	15 (12.7%)	11 (42.3%)	4 (4.3%)	< 0.001
Ophthalmology research interest, N (%)	*Yes*	17 (14.4%)	12 (46.2%)	5 (5.4%)	< 0.001
Ophthalmology clinical elective participation, N (%)	*Yes*	6 (5.1%)	6 (23.1%)	0 (0%)	< 0.001
Ophthalmology clinical elective interest, N (%)	*Yes*	19 (16.1%)	12 (46.2%)	7 (7.6%)	< 0.001
Intent to apply to ophthalmology residency, N (%)	*Yes*	10 (8.5%)	9 (34.6%)	1 (1.1%)	< 0.001
Any interest in working with underserved populations at med school entry, N (%)	*Yes*	73 (76.8%)	19 (79.2%)	54 (76.1%)	> 0.99
Current interest in career working with underserved populations, N (%)	*Yes*	55 (46.6%)	14 (53.8%)	41 (44.6%)	0.51
Service involvement with underserved populations (excl ViSION), N (%)	*Yes*	59 (50%)	17 (65.4%)	42 (45.7%)	0.12
Any interest in service with underserved populations (excl ViSION), N (%)	*Yes*	70 (59.3%)	18 (69.2%)	52 (56.5%)	0.27

^a^*P*-values generated using Fisher’s exact Chi-square test comparing ViSION volunteers to non-volunteers

Overall interest in a career working with underserved populations or in volunteering with community service groups other than ViSION was high and similar between ViSION volunteers and non-volunteers (Table 2).

**Fig. 2:**
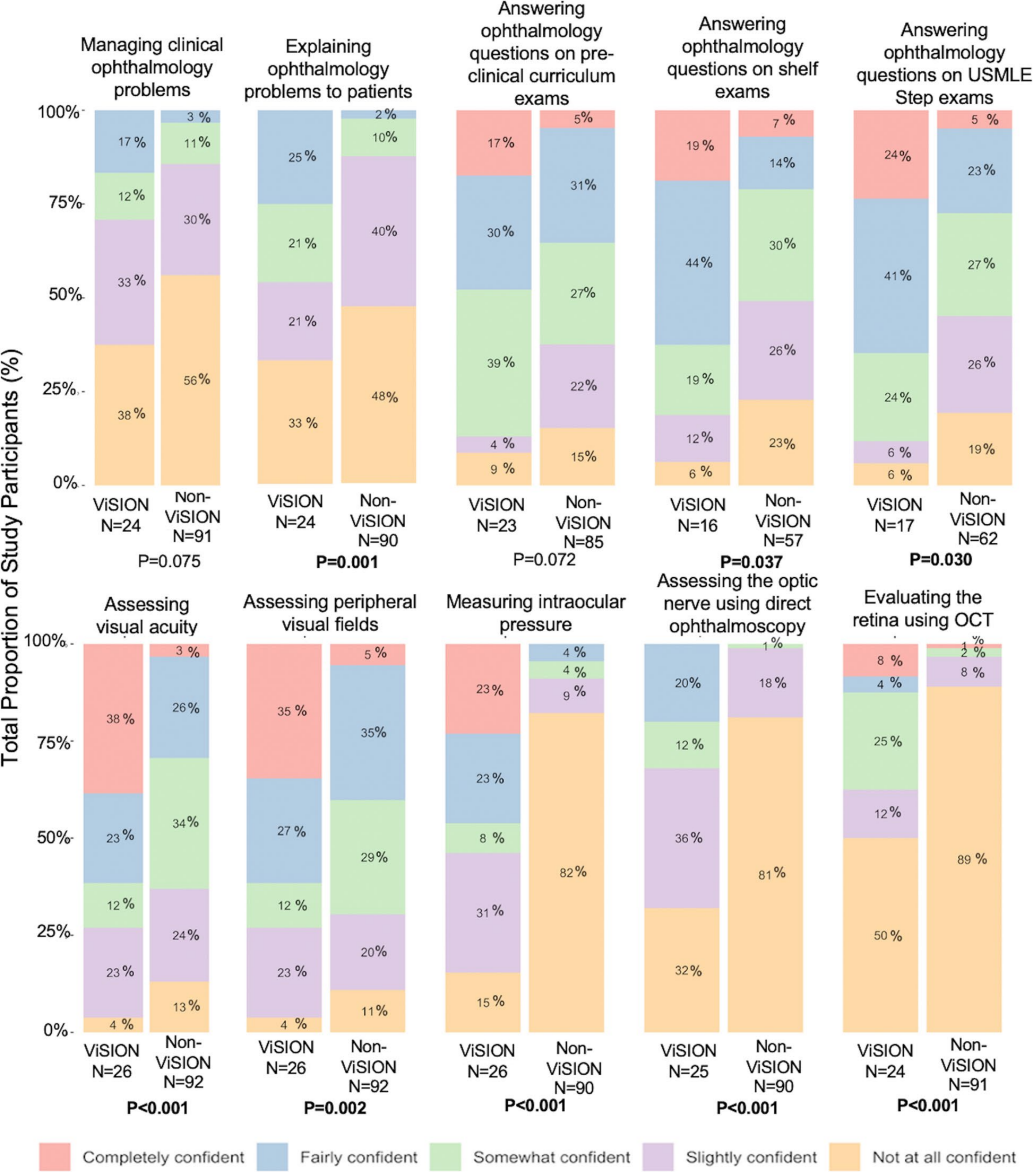
Survey participant confidence with ophthalmology clinical skills and exam content. Stacked bar graphs depict the percentage of respondents that answered each question as indicated in the color-coded legend. Respondents are divided according to whether or not they had volunteered in the JHU-SOM ViSION Program. P-values indicate results of statistical testing using Fisher’s exact Chi-square test

In general, ViSION volunteers reported greater confidence than non-volunteers in multiple aspects of ophthalmology, especially ophthalmology clinical skills (Fig. 2). ViSION volunteers were more confident answering ophthalmology questions on clinical shelf exams (63% fairly or completely confident vs 21% of non-ViSION volunteers, *p* = 0.037) and USMLE step exams (65% vs 28%, *p* = 0.030). ViSION volunteers were also more likely to be fairly or completely confident explaining ophthalmology problems and treatments to patients (25% vs. 2%, *p* = 0.001), assessing visual acuity (61% vs. 29%, *p* < 0.001), measuring intraocular pressure using rebound tonometry (46% vs. 4%, *p* < 0.001), assessing the optic nerve using direct ophthalmoscopy (20% vs. 0%, *p* < 0.001), and evaluating the retina using ocular coherence tomography (12% vs. 1%, *p* < 0.001). Volunteers were not significantly more confident answering ophthalmology questions on pre-clinical curriculum exams or managing ophthalmology problems in a clinic.

Most ViSION volunteers attributed at least some of their ophthalmology-related interests, involvement, and confidence to their experiences with ViSION. In particular, over 60% of ViSION volunteers who conducted or intended to conduct ophthalmology-related research or participated in an ophthalmology clinical elective, and 86% of those who intended to apply to ophthalmology residency programs indicated that their involvement with ViSION influenced these extracurricular and career interests (Supplemental Figure S2). Additionally, the majority of ViSION volunteers who were at least “somewhat confident” performing the ophthalmology clinical skills that were queried in the survey attributed at least some of their confidence to their involvement with ViSION (Supplemental Figure S3). In contrast, only a minority of ViSION participants attributed their confidence answering ophthalmology content on written exams to their experiences with ViSION.

### Qualitative survey

Eleven ViSION volunteers responded to the optional free-form responses regarding their motives for joining, continuing, and terminating participation with ViSION. Five (45%) indicated pre-existing interest in ophthalmology, and 3 (27%) indicated pre-existing interest in applying to ophthalmology residency programs. Exemplary quotes may be found in Supplementary Table S1. Among these respondents, the most common reason for joining ViSION was the desire to “help out in the community” by “perform[ing] service that would actually help patients and link them to care and resources.” Other common motives for joining included wanting to learn more about ophthalmology, practice clinical skills, and be actively involved in patient care. As students continued their involvement with ViSION, their enthusiasm for community service and ophthalmology grew. In particular, students were impressed by the multi-faceted benefits of ViSION, including having “a tremendous impact on our Baltimore community,” “learning more about the social determinants of health,” “gaining a better understanding of common ophthalmologic diseases,” “teaching…younger students to become excited about ophthalmology,” and “getting to work with wonderful faculty.” The primary reason students terminated their involvement with ViSION was time constraints as they transitioned to clinical clerkships.

### Qualitative interviews

Eight students participated in the one-on-one interviews. Four (50%) were female. Four (50%) identified as Asian, 3 (37.5%) as White, and 1 (12.5%) as Black. Three (37.5%) were in their preclinical years and 5 (62.5%) were in their clinical years. All except 1 were current or past ViSION leadership board members. Six (75%) were involved with ViSION for 1–3 years, 1 (12.5%) was involved for less than a year, and 1 (12.5%) for more than 5 years. At the time of the interviews, participants had attended between 3 and greater than 10 screening events (median: 8 events; IQR: 3 to 10). Subthemes were grouped under four main themes: motives, educational value within ophthalmology, educational value outside of ophthalmology, and impact on career. Exemplary quotes for each subtheme may be found in Table 3.

**Table 3:** Themes, subthemes, and exemplary quotes highlighting ViSION volunteers’ experiences and perceptions regarding the impact of ViSION participation on their medical education

Themes^a^	Exemplary Quotes^b^	
**Motives for joining ViSION**		
* Exploring interest in ophthalmology*	“I was interested in ophthalmology and I thought it would be a good way to confirm or deny, if I was interested. And secondly, if I was interested, I knew it would give me, like, good skills that would be useful for my career.”	“I would say the primary reason was because it was related to ophthalmology… I got to practice my skills in the ophthalmic exam and I got to learn more about the field. And those were really meaningful…as a first year med student [since] you don't get to do a whole lot in general that's clinically relevant so [ViSION] was something unique there.”
* Service*	“…it's not just a program that's doing a health fair and screening individuals for vision problems but there is a clear continuity of follow up that we try for all patients…I thought it was really special that patients could get free glasses if they wanted from Wilmer, they could get pro bono care if they didn't have health insurance, and there was even a fund for patients to have surgery if they needed that… I was like, ‘Wow, that's really cool. I want to be involved in something like this.’”	“The main motivation was just really being—being able to be a part of the community that we are currently living in, and doing something that is impactful, not only for our own education but for the people that we serve.”
**Educational value within ophthalmology**		
* Active engagement with ophthalmology*	“I didn't know anything about this equipment, before doing [ViSION]. So I think it really helped like to hear about the different tools that are used… and then using that knowledge…going into for example [the neurology curriculum], like where they were talking about using the OCT to look at the thickness of the retina, to look at the different layers. Also, just like peripheral fields, like [for] things like that…if I didn't go through [ViSION]…, I feel like it would have been really hard for me to understand what they were talking about. So I think it did give me a leg up for [the neurology curriculum]…” “As you continue to go to these events, you start piecing the different puzzles together, [such as how] visual acuity loss in the setting of high intraocular pressure with some fundoscopic changes [is a] hallmark for glaucoma…[that understanding] evolves over time…and I think that was very useful and unique about [ViSION] that you don't necessarily get to appreciate in just a classroom setting.”	“Because when you're shadowing or when you're on your elective, …a lot of the times, most of the role is the attending or the resident who's performing the surgery or seeing the patient in the clinic, and you can jump in, when it's like practical for you to do so, but you really can't have as active of a role in the ophthalmology clinic or OR so I feel like [ViSION] really allows you to take ownership of your like little station, or the like 20 patients you're seeing that day, and make sure that what you're seeing and recording and interpreting is correct.” “I do feel like the ophthalmology portion that we get in didactics is quite limited, which is unfortunate…it really was [at ViSION screening events] where we got to dive into [ophthalmology] in person…[and assess] visual acuity, visual fields, [and] intraocular pressures, [perform] the fundoscopic exam, [see] the OCT images, and then [acquire and interpret] all that data, [make] something meaningful out of it, and [learn about] the disease process [with the help of] the faculty mentors…”
* Exposure to faculty*	“I think it's so good to see role models like our own faculty, showing up and actually like dedicating their time, and like that sets precedence for like the next generation of people who are going to be in that position.”	“I think it was really interesting to see the unique differences that different attendings brought, so of course, some of the glaucoma people of course offered a lot of insights on ‘Hmm, this visual field looks a little bit weird. Let's do a second one instead.’ Whereas some of the retina people were really, really helpful in looking at some of the OCTs and saying, ‘You know, that looks really strange. I actually would want to see them in my clinic.’ And so it was really cool to have people from a variety of different subspecialties.”
**Educational value outside of ophthalmology**		
* Early patient interaction*	“…in terms of rotations, I think just being able to have experiences with patients really helped… you are talking to the patients and you're figuring out what their main worries are…a lot of that is very applicable to an actual rotation, especially the physical exam portion like you have to explain exactly…what the patient needs to do, and ophthalmology is really complicated so your instructions have to be really clear, and so that skill also helped in rotations when you're trying to get [patients] to like, follow a neuro exam or something and you don't really know how to explain it to them but you've already had experience with like translating the [ophthalmic] exam to them so that was pretty helpful.” “This is one of the only student groups where you can actually learn a new skill and work…in a medical setting with patients. [I’ve seen other groups, where] you can do blood pressure testing, but it's not the same, I've been doing that since I was…17. But this was different. [With ViSION, I was] using a physical exam and diagnostic kind of reasoning for the first time, and…doing it in a volunteer setting, and that was just so unique.”	“So it really, I think, was helpful just learning how to talk to people from a different walk of life about their medical issues and trying to get personal information. And then there was also the aspect of dealing with people who had vision impairment—in my own life that's not something I've really dealt with, so I think just learning how to make it a comfortable and safe environment…for them and how to interact with them, was something that I got out of it.” “If I remember first year, I don't think there were a lot of chances to speak to…real patients…You speak to…standardized patients which is…different…[But with ViSION, I was]…speaking to them and taking their history…When… directing them, I [gained] some experience…explaining to them how to do something…in an understandable way for… a lay person.”
* Meaningful service*	“I felt like from the different organizations, I joined and sort of volunteered with, it felt like it was more for …students… trying to improve our clinical skills, learn how to just talk to patients. But it was really [ViSION] where I felt like, wow, we are actually providing a really helpful service to people in the community, like we get to go out to the community, do these amazing screenings, identify all this crazy pathology because they have basically no access to eye care, and then seeing the faculty actually take these people back to Wilmer, being able to follow up with those patients. I thought that was just extraordinary…” “I would say the most valuable part is being able to see a patient who would not have normally gotten seen and screened for an ophth[almology] problem, and then being able to get them the…clinic appointment that they need…[T]hat value… that rewarding feeling you don't really get [in any other setting]…at least in your first and second year because you're really not seeing that many patients…”	“I was able to go to a local like retirement center a couple blocks from Hopkins and like, see where patients live, and that was very important, because it's much different from any experience I've ever had.” “The most unique thing was really serving an underserved population. I guess I was pretty sheltered before it and it was really eye opening…to work with people [for whom] literally the only health care they got at all during that year was seeing [ViSION]. So I think that in terms of opening my eyes to the needs that exist and kind of disparities in Baltimore that was something I wouldn't have necessarily gotten in any other way. Because when you're in the clinic you're only seeing patients who go to clinic. Whereas when you're in…a housing facility in Baltimore, you're getting a lot of people who probably wouldn't otherwise see anybody. I think that was the most unique part.”
* Impact of leadership*	“…when you're on the other end of it, …it just seems pretty straightforward and like seamless and like, easy to do, but when you're actually planning [screening events], it's a lot more difficult and there's a lot of things that you have to think about… you can do even more in the position of being on the board in terms of like helping future students by, and helping future patients by different initiatives that you can put forward.”	“It was always either at the training sessions where you're teaching, or actually at the events when you're teaching the students to, you know, review how to use each of the different devices, what the actual measurements, or values mean, when we should refer, what are the cut offs. So yeah, you're constantly teaching, and I think that's great, I think that's a fantastic part.”
**Impact on career**		
* Increased interest in ophthalmology*	I met my research mentor at one of our screenings, and I worked with him to this day almost four and a half years later. A lot of the work I do right now with [him], is about increasing accessibility and affordability of ophthalmic imaging technology. So I think it had a very direct impact on my research interest too.” “I will certainly say that it wasn't [ViSION] that was…the tipping point for my decision [to go] into ophthalmology, [but] it was definitely a contributing component….I loved the fact that, [ViSION] taught me that there's this huge public health dimension in ophthalmology, that you can actually do this kind of work with the community… But for me personally… basic science vision research was [also] a huge factor.”	“I think it did confirm that I was interested in ophthalmology, and it also kind of helped me decide what area of ophthalmology I was interested in, like, I just loved, looking at the back of the eye [during screenings]. That was like one of my favorite screening days, so I was like “oh maybe glaucoma”, and I ended up doing a research project in glaucoma that summer.”
* Interest in public health*	“When I'm hopefully a resident, …I would want to be the person who still like stays involved in those activities and organizations, like want to teach the med students, get them like excited by ophthalmology, do good work with the community, get them involved into the resident clinic, see them later when I'm a fellow or faculty member, so yeah I think that's going to be a long term kind of thing, and I do think that [ViSION] helped shape that, for sure.” “[ViSION] really was informative for me personally, because it showed me how ophthalmologists can serve those who are less fortunate. There's a perception that I've encountered of surgical subspecialties in general, that it is difficult for them to make [an] impact in those types of communities. And I saw that was definitely not the case…I had mentors and role models and experience to show me that was definitely feasible…That's something I envision for my future career, and it's something that I [now] know I can achieve in a career in ophthalmology.”	“[ViSION] really, I think, for the first time made me interested in kind of public health and serving underserved populations… from the engineering background, …you work in high tech by working with people who have the money to pay for it. But I think I found through [ViSION] I learned about this whole other side of medicine and the whole interest I didn't know I had…on my rotations, it made me a lot more focused on like social determinants of health… I'm doing a master's in BME right now, and one of my projects is related to hearing health, and I've really taken that project in like a community health direction specifically to… help deliver hearing care to underserved communities in Baltimore because I had such a rewarding experience with [ViSION]…I can definitely draw a direct line between that and what I'm doing now.” “I would love in my future career to be a mentor for a group like this… there's a special role for the students being the primary actors or the primary caregivers in this situation, there's a lot for them to learn and to gain from that.”

^a^Themes and subthemes were generated using inductive thematic analysis

^b^Quotes obtained through one-on-one interviews with 8 ViSION volunteers

#### Motives for joining ViSION

##### Exploring interest in ophthalmology

A majority (*n* = 6, 75%) of participants were already interested in ophthalmology prior to joining ViSION, either from personal or undergraduate experiences, and thus joined the program to gain further exposure, confirm or refute their interest in the field, and gain clinical skills in ophthalmology.

##### Service

Engaging in meaningful service and impacting their community was the most common secondary motive for joining (*n* = 5, 62.5%), although it was a primary motive for 2 volunteers (25%).

#### Educational value within ophthalmology

##### Active engagement with ophthalmology

Participants reported that volunteering with the program helped them become familiar with recognizing and interpreting ophthalmic pathology through hands-on experience and application.

“As you continue to go to these events, you start piecing the different puzzles together, [such as how] visual acuity loss in the setting of high intraocular pressure with some fundoscopic changes [is a] hallmark for glaucoma…[that understanding] evolves over time…and I think that was very useful and unique about [ViSION] that you don't necessarily get to appreciate in just a classroom setting.”

While this knowledge did not translate directly into the material tested on pre-clinical or board exams, participants felt that participation in ViSION facilitated accelerated learning of ophthalmology content in didactics or reinforced and increased confidence in the pre-clinical ophthalmology curriculum. Third- and fourth-year medical students noted that this exposure also provided an enhanced fund of knowledge when they entered clinical rotations.

“I would carry around [the direct ophthalmoscope] on my medicine wards…[a]nd I would actually do [my own exam] on any patient who had any issue with vision …and the team actually really appreciated that.”

In terms of clinical skills, participants reported that they became more comfortable performing physical exams, operating equipment, interpreting exam findings, and translating findings to patients. They felt these skills were valuable preparation for a future ophthalmology career. Participants often highlighted the uniqueness of the opportunity of learning to use equipment and gain technical skills in ophthalmology. Many participants noted that the exposure provided by ViSION was particularly valuable given the limited exposure or time spent on ophthalmology content or skills in medical school.

“I do feel like the ophthalmology portion that we get in didactics is quite limited, which is unfortunate…it really was [at ViSION screening events] where we got to dive into [ophthalmology] in person...[and assess] visual acuity, visual fields, [and] intraocular pressures, [perform] the fundoscopic exam, [see] the OCT images, and then [acquire and interpret] all that data, [make] something meaningful out of it, and [learn about] the disease process [with the help of] the faculty mentors…”

The program provided an established, structured avenue to explore ophthalmology early on for both students interested in ophthalmology and those undecided on specialty. Additionally, participants felt that volunteering with the program provided active engagement with the field, which was unique compared to other ophthalmology opportunities in medical school where students often held passive roles in the clinic and the operating room, including on the ophthalmology clinical elective.

##### *Exposure to faculty*

Participants reported that they enjoyed working alongside ophthalmology faculty who volunteered at the screenings and provided teaching and guidance. By volunteering with students, participants noted that these faculty served as role models for maintaining a spirit of service as attending physicians. Many participants also commented on the benefits of connecting to and networking with ophthalmology faculty through the program. Some even identified research projects and long-term mentors through the process.

#### Educational value outside of ophthalmology

##### *Early patient interaction*

Participants emphasized the opportunity provided by the program to directly interact with patients early on in medical school, including taking histories and explaining physical exam maneuvers as first-year medical students, when opportunities to work with patients are often limited to standardized settings or shadowing.

“If I remember first year, I don't think there were a lot of chances to speak to…real patients…You speak to…standardized patients which is…different…[But with ViSION, I was]…speaking to them and taking their history…When… directing them, I [gained] some experience…explaining to them how to do something…in an understandable way for… a lay person.”

Some students discussed learning how to work in a dynamic clinic environment during the screenings and building skills in optimizing teamwork and workflow. Many participants also mentioned that, as opposed to other service opportunities or student organizations like tutoring, for example, ViSION allowed students to develop and practice specific clinical skills and be involved in patient care for particularly diverse, underserved communities.

“This is one of the only student groups where you can actually learn a new skill and work…in a medical setting with patients. [I’ve seen other groups, where] you can do blood pressure testing, but it's not the same, I've been doing that since I was…17. But this was different. [With ViSION, I was] using a physical exam and diagnostic kind of reasoning for the first time, and…doing it in a volunteer setting, and that was just so unique.”

##### *Meaningful service*

By screening and linking patients to care that they might not have been able to access otherwise, participants felt that they were able to provide a tangible and impactful service for their local community. In turn, it was fulfilling for participants to give back to their community, and many participants recounted instances with grateful patients. The ability to perform meaningful community service kept volunteers involved in the program. Some participants noted that it was unique for a service organization to provide such comprehensive care, ranging from screening to arranging follow-up for patients.

“I would say the most valuable part is being able to see a patient who would not have normally gotten seen and screened for an ophth[almology] problem, and then being able to get them the…clinic appointment that they need…[T]hat value… that rewarding feeling you don't really get [in any other setting]…at least in your first and second year because you're really not seeing that many patients...”

Through the program, participants reported gaining a deeper appreciation of service and community engagement and learning more about their own communities. For some, volunteering with the program was their first exposure to working with diverse, underserved communities, public health work, and social determinants of health.

“The most unique thing was really serving an underserved population. I guess I was pretty sheltered before it and it was really eye opening…to work with people [for whom] literally the only health care they got at all during that year was seeing [ViSION]. So I think that in terms of opening my eyes to the needs that exist and kind of disparities in Baltimore that was something I wouldn't have necessarily gotten in any other way. Because when you're in the clinic you're only seeing patients who go to clinic. Whereas when you're in…a housing facility in Baltimore, you're getting a lot of people who probably wouldn't otherwise see anybody. I think that was the most unique part.”

##### *Impact on leadership*

Participants who were on the leadership board spoke of additional responsibilities, such as quality improvement initiatives, delegating tasks among leaders, coordinating events, and logistics. A major responsibility involved training other volunteers and incoming student leaders. They also reported greater exposure to ophthalmology and increased proficiency with the equipment, as a result of longer, sustained involvement in the organization and teaching other volunteers. Student leaders also had increased interactions with faculty and deeper service involvement from their role organizing screening events, and thus, a greater sense of reward.

#### Impact on career

##### Increased interest in ophthalmology

Overall, volunteering with ViSION increased interest in ophthalmology regardless of a volunteer’s pre-existing interest in the field. In particular, the opportunity to perform physical exams, use equipment, and network with faculty facilitated increased interest. For many, ViSION illustrated that ophthalmologists can be involved in public health, which further increased their interest in the field. ViSION complemented volunteers’ other pursuits, including research projects, clinical encounters (shadowing, elective), and the ophthalmology student interest group, to shape their interests in ophthalmology.

“I will certainly say that it wasn't [ViSION] that was…the tipping point for my decision [to go] into ophthalmology, [but] it was definitely a contributing component….I loved the fact that [ViSION] taught me that there's this huge public health dimension in ophthalmology, that you can actually do this kind of work with the community… But for me personally… basic science vision research was [also] a huge factor.”

Participants reported that involvement in ViSION helped them discover new clinical interests, whether it be in specific ophthalmic subspecialties or teaching/mentoring. Research projects stemmed out of ViSION, either by networking with faculty at the screening, quality improvement projects for ViSION, or by inspiring the subject area.

“I met my research mentor at one of our screenings, and I worked with him to this day almost four and a half years later. A lot of the work I do right now with [him], is about increasing accessibility and affordability of ophthalmic imaging technology. So I think it had a very direct impact on my research interest too.”

##### *Interest in public health*

For many participants, ViSION inspired or further reinforced their desire to pursue a public health component to their career, alleviate disparities, or work with underserved communities. Participants emphasized experiencing the intersection of ophthalmology and public health and the importance of this overlap in their future careers.

“[ViSION] really was informative for me personally, because it showed me how ophthalmologists can serve those who are less fortunate. There's a perception that I've encountered of surgical subspecialties in general, that it is difficult for them to make [an] impact in those types of communities. And I saw that was definitely not the case…I had mentors and role models and experience to show me that was definitely feasible…That's something I envision for my future career, and it's something that I [now] know I can achieve in a career in ophthalmology.”

Participants noted that they wanted to pursue similar initiatives and programs in residency and as future attending physicians, in order to continue doing service work, but also to mentor students and expose them to ophthalmology.

“I would love in my future career to be a mentor for a group like this… there's a special role for the students being the primary actors or the primary caregivers in this situation, there's a lot for them to learn and to gain from that.”

## Discussion

In this mixed-methods cross-sectional study, we found that a greater proportion of students participating in a community vision screening initiative were interested in ophthalmology and confident with their ophthalmology skills than students who did not participate. Responses to survey questions and individual interviews revealed that volunteers attributed at least some of their ophthalmology skills and desire to pursue ophthalmology and public health work to their ViSION experiences. It is noteworthy that only one third of ViSION volunteers had applied to or intended to apply to ophthalmology residency. Moreover, only half of volunteers planned to enroll in the ophthalmology clinical clerkship. For the two thirds of volunteers planning to enter other specialties, the ViSION program provided experiences that were unlikely to have occurred throughout the entirety of their training and therefore may have served to fill an important educational gap.

A number of community service experiences in ophthalmology have been described in the literature [8–10], but evidence regarding their impact on medical education is limited. One study demonstrated that student volunteers attained better ophthalmology knowledge scores and ophthalmoscopy skills relative to students and residents who did not volunteer [12]. Another study described the benefits that such a program provides for medical students by listing the opportunities it provided (e.g., slit lamp training, guidance while performing comprehensive eye exams)[9], while a recent mixed-methods study highlighted the impact of a near-peer teaching program on students’ confidence and aptitude teaching ophthalmology skills to other students [11]. Our study adds a new perspective to the existing literature by combining quantitative analysis of student-reported interests and confidence with in-depth semi-structured one-on-one interviews to highlight the myriad ways in which community service experiences in ophthalmology may enhance medical education.

Given the voluntary nature of ViSION, volunteers’ increased interest in pursuing ophthalmology as a career or research endeavor may be due to confirmation bias (i.e. students with greater baseline interest in ophthalmology are more likely to engage with ophthalmology extracurricular activities). Indeed, in one-on-one interviews, volunteers identified their prior interest in ophthalmology as the primary motivating factor for joining ViSION. However, our interviews suggest a bidirectionality to the association as volunteers also reported that their involvement with ViSION strengthened their interest in ophthalmology and revealed new and exciting aspects of the field, such as by inspiring research projects or providing networking opportunities with faculty. Additionally, the service and public health aspects of ViSION further buttressed volunteers’ interest in ophthalmology, findings that are consistent with previous studies demonstrating increased interest in social justice among individuals engaged in service-learning programs [19, 20]. For all students interested in ophthalmology, regardless of the stimulating factor, ViSION provided a unique opportunity to explore the field and learn about common ocular pathologies in an interactive setting.

Concordant with previous studies demonstrating a decline in undergraduate medical curricular hours in ophthalmology [6], only 1% of non-ViSION volunteers reported even some degree of confidence using the direct ophthalmoscope (Fig. 1). In contrast, ViSION volunteers were significantly more likely than their non-ViSION counterparts to report confidence performing an array of basic eye examination techniques relevant to many non-ophthalmologic specialties, which interview participants contributed heavily to ViSION involvement. Interestingly, even volunteers who also participated in the ophthalmology clinical elective noted that the hands-on aspect of ViSION community screenings was a unique opportunity unparalleled at any other time during their medical school training. This may suggest that the largely lecture-based pre-clinical training in ophthalmology that medical students receive [6] does not adequately prepare students to take on an active role during the clinical elective. Indeed, this is consistent with studies showing that competency-based ophthalmology education involving self-directed learning, peer-assisted learning, and problem-based learning is more effective than curricula that focus solely on content or number of hours spent learning ophthalmology [21–23]. Additionally, in both survey and interview responses, ViSION volunteers reported greater confidence with ophthalmology concepts presented in the curriculum than their non-volunteer counterparts. Participation in extracurricular vision screening service-learning programs provides the benefits of active learning and repeated exposure, which are well-described in the medical education literature, particularly in the context of clerkship shelf exams and USMLE step 1 exams [25–27].

During semi-structured interviews, participants also noted benefits that apply generally outside of ophthalmology, ranging from skill building in patient communication and interaction to leadership. These findings are consistent with previous work both within and outside of ophthalmology that have demonstrated that service activities provide a useful scaffold for medical students to practice working in interprofessional teams [13] and gain experience in clinical peer-to-peer teaching and mentoring [11, 14, 16].

Beyond having a role in ophthalmology-related medical education, these service-learning programs share a common objective of providing eye care to underserved populations in local communities. Previous studies have highlighted the importance of non-ophthalmology service-learning programs for providing students with opportunities to be more civically engaged [13, 14, 16–18] and gain exposure to fields like primary care and social justice [19, 20]. Additionally, such programs positively influence students’ attitudes toward working with underserved populations [28, 29]. Unfortunately, medical students often equate a service-oriented career and work with underserved populations with primary care [29]. Despite being surgical subspecialists, however, ophthalmologists have an important role to play within underserved communities. Student responses to interview questions and open-ended survey questions in the present study demonstrated that the opportunity to be involved in the local community and have significant impact on underserved populations was a major motivating factor for joining and continuing with ViSION. Notably, the public health aspect of ophthalmology surprised a number of students and positively influenced their attitude towards the field. By introducing this aspect of ophthalmology, programs like ViSION may have a long-lasting impact on students’ career trajectories in ophthalmology, such as pursuing public health research and initiatives or practicing in underserved communities. It may also draw students already inclined towards a service-oriented career to ophthalmology. Overall, these combined survey and interview data suggest that vision screening service-learning programs, like ViSION, offer an exciting and effective avenue by which to enhance medical student education in ophthalmology.

## Strengths and weaknesses

This study highlights a promising role for a vision screening service-learning program in medical education. Our results are limited by the inability to randomize medical students to volunteer experiences, the cross-sectional nature of the data, the modest response rate, and a lack of objective measures of ophthalmology knowledge and skills. In addition, we recognize that most interview participants had previously served in ViSION leadership positions. Their views, therefore, may not be fully representative of the less invested ViSION volunteers. However, using purposive sampling to gather the perspectives of the most experienced, and thus informed, volunteers allowed us to explore student perceptions regarding how ViSION influenced their interests and education. In addition, our survey’s qualitative responses were generally in agreement with interviewee responses, and the demographics of our survey population were relatively similar to that of the entire JHU-SOM. Prospective longitudinal studies, including follow-up with students to determine what fraction ultimately pursue careers in public health and ophthalmology, would be helpful to explore the true educational value of community vision screening programs.

## Conclusions

Volunteers in a medical student-led community vision screening program at JHU-SOM had a greater interest and involvement in ophthalmology-related activities and greater confidence with their ophthalmology knowledge and skills. Importantly, they considered their experiences with ViSION to be invaluable in shaping their medical school education in ophthalmology. This is particularly powerful in the setting of scant ophthalmology exposure within medical school curricula nationwide and the lack of confidence in ophthalmology clinical skills within non-ophthalmology medical communities at large. Additionally, ViSION provided an outlet for medical students to explore interests in public health and discover the significant role that surgical subspecialists, and ophthalmologists specifically, may play in community health outreach. Therefore, student-led community vision screening programs have the potential to serve an important adjunctive role in medical school ophthalmology curricula, helping to prepare future ophthalmology residents for further training and non-ophthalmology physicians to recognize basic ophthalmology problems, while also providing a humanitarian service to the local community.

## Supplementary Information

**Additional file 1.** Survey Outline: Start of Block: Part 1 for all current or former JHUSOM students

**Additional file 2.** Interview Outline: One-on-One In-Depth Interview Questions

**Additional file 3.** Interview Codebook

**Additional file 4: Figure S1.** Flow chart of survey responses included in analysis.

**Additional file 5: Figure S2.** Survey participant attributions of ophthalmology-related and service-oriented interests, extracurricular participation, and ophthalmology curriculum engagement to participation in ViSION. Stacked bar graphs depict the percentage of respondents that answered each question as indicated in the color-coded legend. Only respondents who indicated interest or participation with a given activity or who indicated feeling at least “somewhat interested or engaged” (level 3 interest on a 5-point scale) in the ophthalmology curriculum were included.

**Additional file 6: Figure S3.** Survey participant attributions of confidence with ophthalmology clinical skills and exam content to participation in ViSION. Stacked bar graphs depict the percentage of respondents that answered each question as indicated in the color-coded legend. Only respondents who volunteered in the JHU-SOM ViSION Program and indicated feeling at least “somewhat confident” (level 3 confidence on a 5-point scale) performing a given skill or answering exam questions were included.

**Additional file 7: Table S1. **ViSION volunteers’ motives for joining, continuing, and terminating ViSION involvement based on survey responses.

## Data Availability

The datasets supporting the conclusions of this article are available in anonymized format from the corresponding author on reasonable request. The datasets generated during and analyzed during the current study are not publicly available as participants were assured that their individual responses would not be disclosed. However, de-identified, consolidated data are available from the corresponding author upon reasonable request.

## Abbreviations

AUPO: Association of University Professors of Ophthalmology
JHU-SOM: Johns Hopkins University School of Medicine
OCT: Optical coherence tomography
USMLE: Unites States Medical Licensing Examination
ViSION: **Vision Screening In Our Neighborhoods**
