# Supplementary material for: Demographics, clinical interests, and ophthalmology skills confidence of medical student volunteers and non-volunteers in an extracurricular community vision screening service-learning program
Source: BMC Med Educ. 2022 Mar 4;22:143. doi: 10.1186/s12909-022-03194-0 (PMC8894556; doi:10.1186/s12909-022-03194-0)
Supplement: Supplementary file 1 — Additional file 1. Survey Outline: Start of Block: Part 1 for all current or former JHUSOM students [file 12909_2022_3194_MOESM1_ESM.docx]

**Survey Outline:**

Start of Block: Part 1 for all current or former JHUSOM students

intro We are conducting an educational program evaluation and education research study (JHM IRB00233696) to understand how medical student experiences during medical school influence their choice of extracurricular activities, elective rotations, and career trajectory. **Please complete this survey only if you are a current student of the Johns Hopkins University School University of Medicine (actively enrolled or on leave while you obtain another degree or engage in research opportunities)**. You will be presented with several questions assessing your extracurricular involvement and career intentions during medical school. This will take **less than 5 minutes**.
You will then be asked if you wish to answer a few free-form questions regarding your participation in the Student Sight Savers Program. **For this part of the survey**, **you must be an active or former member of the Student Sight Savers Program.** The entire survey should take **less than 12 minutes** to complete.
Your participation is voluntary, and your completion of this survey serves as your consent to participate. Your decision of whether or not to participate in this research will not affect employment, education, or training at Johns Hopkins. Your individual responses will be kept completely confidential.**Those who complete the survey are eligible to enter a drawing for one of four $50 Amazon gift cards.** At the end of the survey, you will be provided with a link to a separate form to provide your email address, should you choose to participate in this drawing. Your email address will only be used to distribute your gift card should you be selected as a recipient and will not be linked in any way to your survey responses. Entering in this drawing and providing your email address are not requirements to participate in this study.  The Principal Investigator for this study is Thomas V Johnson III, MD, PhD. You may contact the study team at eburton5@jhmi.edu or johnson@jhmi.edu to learn more about the study.

taken Have you taken this survey before?

- Yes (1)
- No (2)

Display This Question:

If Have you taken this survey before? = Yes

| 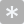 |
| --- |

username_old Please input the same 8 character username that you used last time that you took this survey.

________________________________________________________________

Display This Question:

If Have you taken this survey before? = No

username_new Please input an 8-character memorable username. Next time you take this survey, we will ask you to input the same username, so please record it in a place that you will remember.

________________________________________________________________

gender How do you identify?

- Male (1)
- Female (2)
- Other (3)

race Please specify your race (check all that apply):

- Asian (1)
- American Indian or Alaska Native (2)
- Black or African American (3)
- Native Hawaiian or Pacific Islander (4)
- White (5)
- Other (6) ________________________________________________
- Prefer not to answer (7)

ethnicity Are you of Hispanic, Latino, or Spanish origin?

- Yes (1)
- No (2)

birthyear What is your year of birth?

▼ 1975 (1) ... 2001 (27)

medical_training What is your current stage of medical training?

- MS1 (1)
- MS2 (2)
- MS3 (3)
- MS4 (4)
- MSTP research year 1 (5)
- MSTP research year 2 (6)
- MSTP research year 3 (7)
- MSTP research year 4 (8)
- MSTP research year 5+ (9)
- Gap year (10)
- Other (11) ________________________________________________

ophtho_interest_init Did you have any particular interest in ophthalmology upon starting medical school?

- Yes (1)
- No (2)
- Unsure (3)

ophtho_interest_curr Do you currently plan to pursue a career in ophthalmology?

- Yes (1)
- No (2)
- Unsure (3)

underserved_initial “The Department of Health and Human Services (HHS) characterizes underserved, vulnerable, and special needs populations as communities that include members of minority populations or individuals who have experienced health disparities… Underserved populations include consumers who share one or more of the following characteristics. • Receive fewer health care services. • Encounter barriers to accessing primary health care services (e.g., economic, cultural, and/or linguistic). • Have a lack of familiarity with the health care delivery system. • Face a shortage of readily available providers.” 


  **Did you have any particular interest in working (clinically or otherwise) specifically (but not necessarily exclusively) with underserved populations upon starting medical school?**

- Yes (1)
- No (2)
- Unsure (3)

underserved_current Do you currently plan to pursue a career (clinical or otherwise) involving care specifically (but not necessarily exclusively) for underserved populations?

- Yes (1)
- No (2)
- Unsure (3)

Display This Question:

If Do you currently plan to pursue a career (clinical or otherwise) involving care specifically (but... = Yes

career_intentions Briefly describe your intentions

________________________________________________________________

service_underserved During medical school, have you volunteered with any groups besides the Student Sight Savers Program focused on serving, advocating for, or educating underserved populations? If so, which ones?

- Yes (1) ________________________________________________
- No (2)
- Plan to (3)

Display This Question:

If During medical school, have you volunteered with any groups besides the Student Sight Savers Prog... = Yes

service_motivations Which factors motivated you to join the groups that you listed?

________________________________________________________________

ophtho_research Have you ever participated in ophthalmology-related research?

- Yes (1)
- No (2)
- Plan to (3)

Display This Question:

If Have you ever participated in ophthalmology-related research?  = Yes

ophto_research_spec In what type(s) of ophthalmology research have you participated (select all that apply)?

- Public Health (1)
- Basic Science (2)
- Clinical (3)
- Ethics (4)
- Education (5)
- Other (please specify) (6) ________________________________________________

ophtho_elective During medical school, have you completed or attempted to complete an elective rotation in ophthalmology?

- Yes (1)
- No (2)
- Plan to (3)

Display This Question:

If During medical school, have you completed or attempted to complete an elective rotation in ophtha... = Yes

ophtho_elective_spec In which ophthalmology specialty did you complete or attempt to complete an elective rotation (select all that apply)?

- Cornea (1)
- Retina (2)
- Glaucoma (3)
- Pediatrics (4)
- Oculoplastics (5)
- Neuro-ophthalmology (6)
- Uveitis (7)
- Other (please specify) (8) ________________________________________________

residency Have you or are you planning to apply for an ophthalmology residency position?

- I have applied to ophthalmology residency (1)
- I plan to apply to ophthalmology residency (2)
- No, I have applied to or plan to apply to: (3) ________________________________________________
- No, I do not know which residency I will apply to (4)

confidence_skill Rate how **confident** you feel now in your ability to execute the following skills:

|  | Not at all confident (1) | Slightly confident (2) | Somewhat confident (3) | Fairly confident (4) | Completely confident (5) |
| --- | --- | --- | --- | --- | --- |
| Managing, triaging, or understanding ophthalmology problems encountered in a clinical setting (1) |  |  |  |  |  |
| Describing and explaining eye problems and treatments to patients (2) |  |  |  |  |  |
| Assessing a patient’s visual acuity (3) |  |  |  |  |  |
| Assessing a patient’s peripheral visual field (4) |  |  |  |  |  |
| Measuring a patient’s intraocular pressure (5) |  |  |  |  |  |
| Estimating a patient’s cup-to-disc ratio with fundoscopy (6) |  |  |  |  |  |
| Evaluating a patient’s retina using an OCT (7) |  |  |  |  |  |

confidence_exams Rate how **confident** you feel/felt in your understanding of ophthalmology-related content as encountered in the following settings:

|  | Not at all confident (1) | Slightly confident (2) | Somewhat confident (3) | Fairly confident (4) | Completely confident (5) | N/A (6) |
| --- | --- | --- | --- | --- | --- | --- |
| MS1/MS2 curricular exams (1) |  |  |  |  |  |  |
| Clinical shelf exams (2) |  |  |  |  |  |  |
| USMLE step exams (3) |  |  |  |  |  |  |

engagement Estimate your level of **interest** and **engagement** with the ophthalmology-related material during your medical school curriculum:

- Actively Uninterested and disengaged (1)
- Somewhat Uninterested or disengaged (2)
- Felt neutral (3)
- Somewhat interested or engaged (4)
- Extremely interested and engaged (5)
- N/A (6)

engagement_comp **Compared with other content** learned during your medical school curriculum, rate your level of **interest** in/**engagement** with the ophthalmology-related material:

- Much less interested or engaged (1)
- Somewhat less interested or engaged (2)
- The same (3)
- Somewhat more interested or engaged (4)
- Much more interested or engaged (5)
- N/A (6)

oig During medical school, have you participated in the Ophthalmology Interest Group?

- Yes (1)
- No (2)

sssp During medical school, have you participated in the Student Sight Savers Program?

- Yes (1)
- No (2)

Skip To: End of Survey If During medical school, have you participated in the Student Sight Savers Program? = No

End of Block: Part 1 for all current or former JHUSOM students

Start of Block: Part 2 for SSSP Participants

sssp intro The following questions will ask you more about your experience with the Student Sight Savers Program. Please answer openly and truthfully.

sssp_join When did you join the Student Sight Savers Program (SSSP)?

- MS1 (1)
- MS2 (2)
- MS3 (3)
- MS4 (4)
- MSTP research year 1 (5)
- MSTP research year 2 (6)
- MSTP research year 3 (7)
- MSTP research year 4 (8)
- MSTP research year 5+ (9)
- Gap Year (10)
- Other (11) ________________________________________________

sssp_duration For how long were you/have you been involved with the SSSP?

- <1 year (1)
- between 1 and 2 years (2)
- between 2 and 3 years (3)
- between 3 and 4 years (4)
- between 4 and 5 years (5)
- >5 years (6)

sssp_motives Why did you join SSSP? (RANK ALL THAT APPLY: **1 indicates most important**, higher numbers indicate less important)

______ Interested in pursuing a career in ophthalmology (1)

______ Interested in learning more about ophthalmology (2)

______ Interested in primary care or preventative medicine (3)

______ Wanted more direct patient contact (4)

______ Strengthen interpersonal and/or clinical skills (5)

______ Organized community service (6)

______ Immersion with the Baltimore community (7)

______ Strengthen resume (8)

______ For fun (9)

______ Friend convinced me (10)

______ Other (11)

sssp_motives_text Please elaborate on your reason(s) for joining the Student Sight Savers Program.

________________________________________________________________

sssp_continued Why did you continue your involvement with SSSP? (RANK ALL THAT APPLY: **1 indicates most important**, higher numbers indicate less important)

______ Interested in pursuing a career in ophthalmology (1)

______ Interested in learning more about ophthalmology (2)

______ Interested in primary care or preventative medicine (3)

______ Wanted more direct patient contact (4)

______ Strengthen interpersonal and/or clinical skills (5)

______ Organized community service (6)

______ Immersion with the Baltimore community (7)

______ Strengthen resume (8)

______ For fun (9)

______ Friend convinced me (10)

______ Other: (11)

sssp_continued_text Please elaborate on your reason(s) for continuing your involvement with SSSP (memorable moments are welcomed)

________________________________________________________________

sssp_terminate If you terminated your involvement with the SSSP prior to medical school graduation, why did you do so? (RANK ALL THAT APPLY: **1 indicates most important**, higher numbers indicate less important)

______ No longer interested in ophthalmology (1)

______ Time constraints with pre-clinical work (2)

______ Time constraints with clinical rotations (3)

______ Time constraints studying for USMLE Step 1 (4)

______ Other: (5)

sssp_terminate_text  Please elaborate on your reason for terminating involvement with the SSSP. If you have not terminated your involvement with the SSSP, please type N/A.

________________________________________________________________

sssp_activities In which of the following SSSP activities did you participate? (RANK ALL THAT APPLY: **1 indicates most participation**, higher numbers indicate less participation)

______ Adult community screenings (1)

______ Child school screenings (2)

______ Event planning (3)

______ E-board member (4)

______ Appreciation dinner attendance (5)

______ SSSP/ophthalmology-hosted liver/evening rounds (6)

______ Other: (7)

sssp_activities_text Why did you choose to participate in the particular activities indicated above?

________________________________________________________________

Display This Question:

If In which of the following SSSP activities did you participate? (RANK ALL THAT APPLY: 1 indicates ... [ Adult community screenings ] > 0

adult_screen How many adult screenings did you attend?

________________________________________________________________

Display This Question:

If In which of the following SSSP activities did you participate? (RANK ALL THAT APPLY: 1 indicates ... [ Child school screenings ] > 0

child_screen How many child school screenings did you attend?

________________________________________________________________

sssp_stations_rank Which of the following screening station positions did you hold? (RANK ALL THAT APPLY: **1 indicates most participation**, higher numbers indicate less participation)

______ Registration/Check-out (1)

______ Visual Acuity (Titmus VA analyzer of Snellen wall chart) (2)

______ Peripheral Acuity (FDT) (3)

______ Intraocular Pressure (I-care tonometer) (4)

______ Ocular Coherence Tomography (OCT) (5)

______ Optic nerve cup-to-disc ratio (Panoptic) (6)

sssp_influence Please indicate your agreement with the following:My involvement in SSSP influenced my decision to participate in:

|  | Strongly Disagree (1) | Disagree (2) | Somewhat disagree (3) | Neither agree nor disagree (4) | Somewhat agree (5) | Agree (6) | Strongly agree (7) | N/A (8) |
| --- | --- | --- | --- | --- | --- | --- | --- | --- |
| Ophthalmology research (1) |  |  |  |  |  |  |  |  |
| Ophthalmology elective rotation (2) |  |  |  |  |  |  |  |  |
| Ophthalmology residency (3) |  |  |  |  |  |  |  |  |
| Other extracurriculars involving underserved populations (4) |  |  |  |  |  |  |  |  |

confidence_bc_sssp To what degree do you attribute your confidence level with each of the following skills or exams to your experiences with the SSSP?

|  | Not at all (1) | A small amount (2) | Somewhat (3) | A moderate amount (4) | A great deal (5) | N/A (6) |
| --- | --- | --- | --- | --- | --- | --- |
| Managing, triaging, or understanding ophthalmology problems encountered in a clinical setting (1) |  |  |  |  |  |  |
| Describing and explaining eye problems and treatments to patients (2) |  |  |  |  |  |  |
| Assessing a patient’s visual acuity (3) |  |  |  |  |  |  |
| Assessing a patient’s peripheral visual field (4) |  |  |  |  |  |  |
| Measuring a patient’s intraocular pressure (5) |  |  |  |  |  |  |
| Estimating a patient’s cup-to-disc ratio with fundoscopy (6) |  |  |  |  |  |  |
| Evaluating a patient’s retina using an OCT (7) |  |  |  |  |  |  |
| MS1/MS2 curricular exams (8) |  |  |  |  |  |  |
| Clinical shelf exams (9) |  |  |  |  |  |  |
| USMLE step exams (10) |  |  |  |  |  |  |

engagement_bc_sssp To what degree would you estimate that your experiences with **SSSP contributed** to your level of **interest** in/**engagement** with ophthalmology-related content encountered during your medical school curriculum?

- Not at all (1)
- A small amount (2)
- Somewhat (3)
- A moderate amount (4)
- A great deal (5)
- N/A (6)

suggestions The Student Sight Savers Program would not function without amazing volunteers like you! Please take a moment to provide any ideas or suggestions for how to improve the SSSP?

________________________________________________________________

End of Block: Part 2 for SSSP Participants
