# Supplementary material for: Demographics, clinical interests, and ophthalmology skills confidence of medical student volunteers and non-volunteers in an extracurricular community vision screening service-learning program
Source: BMC Med Educ. 2022 Mar 4;22:143. doi: 10.1186/s12909-022-03194-0 (PMC8894556; doi:10.1186/s12909-022-03194-0)
Supplement: Supplementary file 2 — Additional file 2. Interview Outline: One-on-One In-Depth Interview Questions [file 12909_2022_3194_MOESM2_ESM.docx]

**Interview Outline:**

One-on-One In-Depth Interview Questions

We would like to know more about your involvement in Vision Screening In Our Neighborhoods (ViSION) program, formerly known as the Student Sight Savers Program (SSSP). We are especially interested in the impact you believe it had on your medical education, interest in ophthalmology, and interest in working with underserved populations. We are also interested in learning more about its potential impact on your career choices. This interview will be audio-recorded. Thank you very much for taking the time to share your experience with us.

1. Tell me a little bit about your involvement with ViSION/SSSP.  
   *Use these questions to guide the participant.*
2. When did you first join?
3. How did you decide to join?
4. Are you still involved?
5. What was your main motive behind joining ViSION/SSSP?  
   *Use these questions to guide the participant.*
6. Did you have any interest in ophthalmology upon signing-up to volunteer? In preventative medicine or primary care? In health care disparities?
7. Were you interested in learning more about ophthalmology and eye diseases? Were you looking for clinical exposure and direct patient contact?
8. How do you think your involvement with ViSION/SSSP helped you learn ophthalmology content?
9. How did it impact your knowledge of ophthalmology concepts?
10. How did it impact your performance on ophthalmology-related questions in MS1/MS2 curricular exams, clinical shelf exams, USMLE exams?
11. How did it impact your clinical skills in ophthalmology?
12. How do you think your involvement with ViSION/SSSP shaped your clinical interests? Your research interests?
13. How do you think your involvement with ViSION/SSSP shaped your career goals?  
    *Use these questions to guide the participant.*
14. *Did it influence your specialty choice?*
15. *Did it influence your choice of setting where you’d like to train or practice?*
16. What do you think is the most valuable aspect of volunteering with ViSION/SSSP? How do you think the SSSP volunteering experience could be made more valuable or educational for students?
17. What do you think are some barriers, limitations, or challenges to participating in ViSION/SSSP? How do you think these could be addressed?
18. Do you have any other thoughts about your experience with ViSION/SSSP that we haven’t touched on?
19. Thank you for sharing your thoughts with us today. We really appreciate it.
