# Supplementary material for: Demographics, clinical interests, and ophthalmology skills confidence of medical student volunteers and non-volunteers in an extracurricular community vision screening service-learning program
Source: BMC Med Educ. 2022 Mar 4;22:143. doi: 10.1186/s12909-022-03194-0 (PMC8894556; doi:10.1186/s12909-022-03194-0)
Supplement: Supplementary file 3 — Additional file 3. Interview Codebook [file 12909_2022_3194_MOESM3_ESM.docx]

**Interview Codebook**

| Name | Description |
| --- | --- |
| Barriers |  |
| Limited opportunities | Spots per screening, tasks |
| Logistics | Including transportation |
| Time | Medical students are busy, hard to find a time that works for all |
| Training | Gaps between screenings, barrier to entry if miss training session |
| Benefits | Valuable and/or unique aspects of ViSiON, what they gained |
| Clinical workflow | Working together with peers, teamwork, managing the clinic |
| Early patient interaction and clinical care | Learning how to speak to patients, etc |
| Limited direct patient contact in preclinical |  |
| Educational value |  |
| Greater exposure to content and concepts through hands-on experience |  |
| Ophthalmology skills | Including equipment, exams, etc |
| Exposure to faculty and networking |  |
| Exposure to ophthalmology as a field | For both those interested or not interested in ophthalmology |
| Limited ophthalmology exposure in medical school | This is one of the only exposures/activities, especially early on |
| Service |  |
| Exposure to underserved and learning about the community | Including community resources, social determinants of health |
| Fulfilling | Rewarding, meaningful way to make a difference |
| Important for applications |  |
| Social aspect | Getting to know classmates, fun experience with friends |
| Impact of leadership role |  |
| Deeper service involvement | Learning more about community resources, etc |
| Greater exposure to the field via deeper, sustained involvement |  |
| Responsibility |  |
| Teaching and training others |  |
| Work more closely with faculty |  |
| Impact on career goals and interests |  |
| Helped increase interest in ophthalmology |  |
| Other experiences helped shape interests (including dissuading from ophtho) | In addition to ViSiON |
| Research and clinical |  |
| Underserved communities and public health angle |  |
| Want to pursue similar program in the future | Join similar program as resident, faculty, or help set up this program |
| Interested in being a mentor to students in these programs |  |
| Motive |  |
| Already interested in ophthalmology |  |
| Explore to confirm or deny |  |
| Exposure |  |
| Skills | Including tech, equipment, exams, etc |
| Not initially interested in ophthalmology |  |
| Opportunity for patient interaction | More direct clinical care, etc |
| Primary motive |  |
| Secondary motive |  |
| Service | Looking for service opportunity, working with underserved, etc. |
| Social aspect was helpful | Had friends who were doing it who encouraged involvement |
| Prior interest in ophthalmology |  |
| First hand experience |  |
| Undergraduate experiences |  |
| Suggestions |  |
| Follow-up |  |
| More opportunities | Including varying the stations, more screenings, etc |
| More training and refreshers | Including pre-assigning with instructions, more frequent trainings, etc |
| Other | Anything else |
