# Supplementary figures and images for: Demographics, clinical interests, and ophthalmology skills confidence of medical student volunteers and non-volunteers in an extracurricular community vision screening service-learning program
Source: BMC Med Educ. 2022 Mar 4;22:143. doi: 10.1186/s12909-022-03194-0 (PMC8894556; doi:10.1186/s12909-022-03194-0)

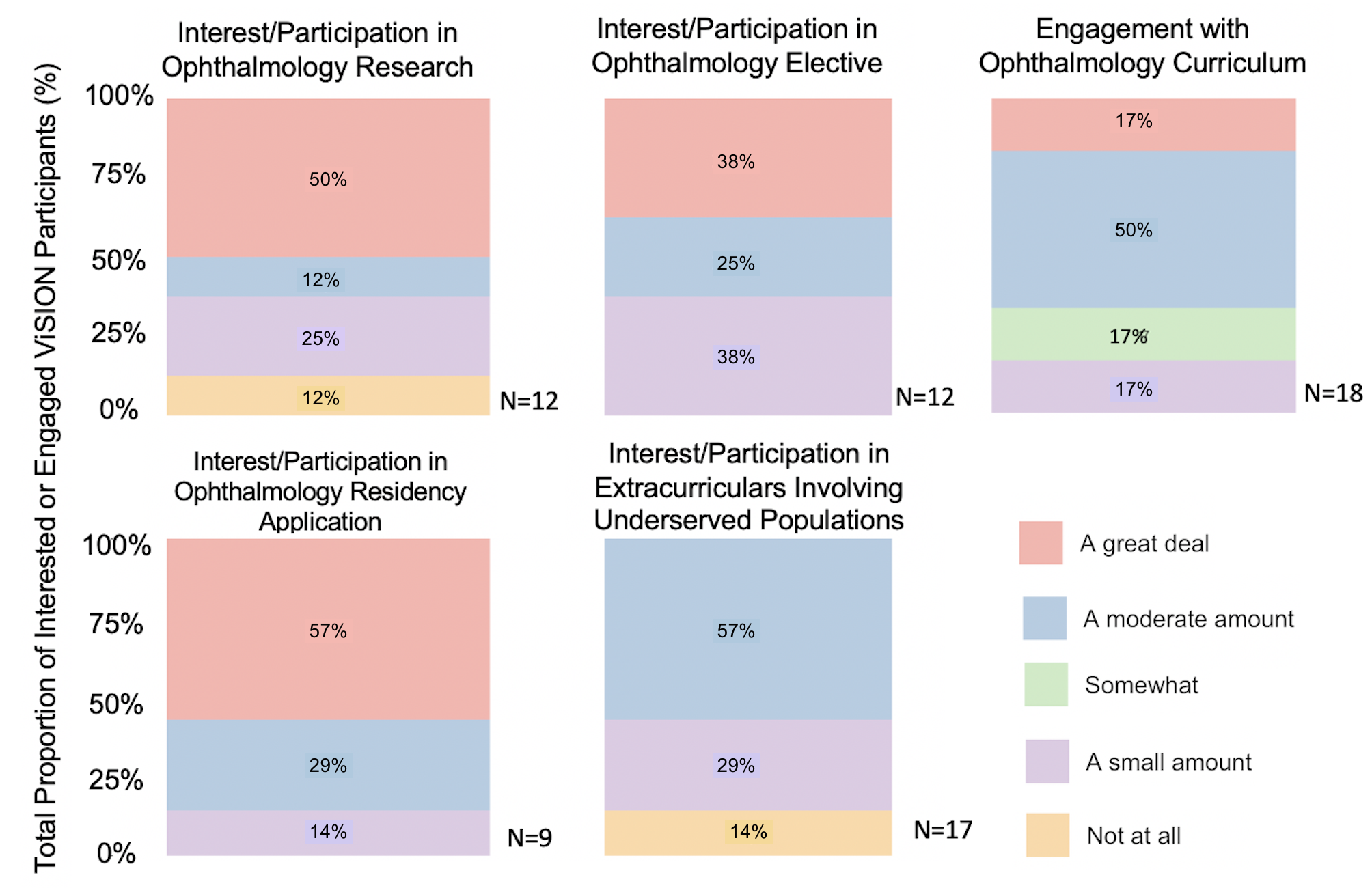

Supplement: Supplementary file 4 — Additional file 4: Figure S1. Flow chart of survey responses included in analysis. [file 12909_2022_3194_MOESM4_ESM.tiff]

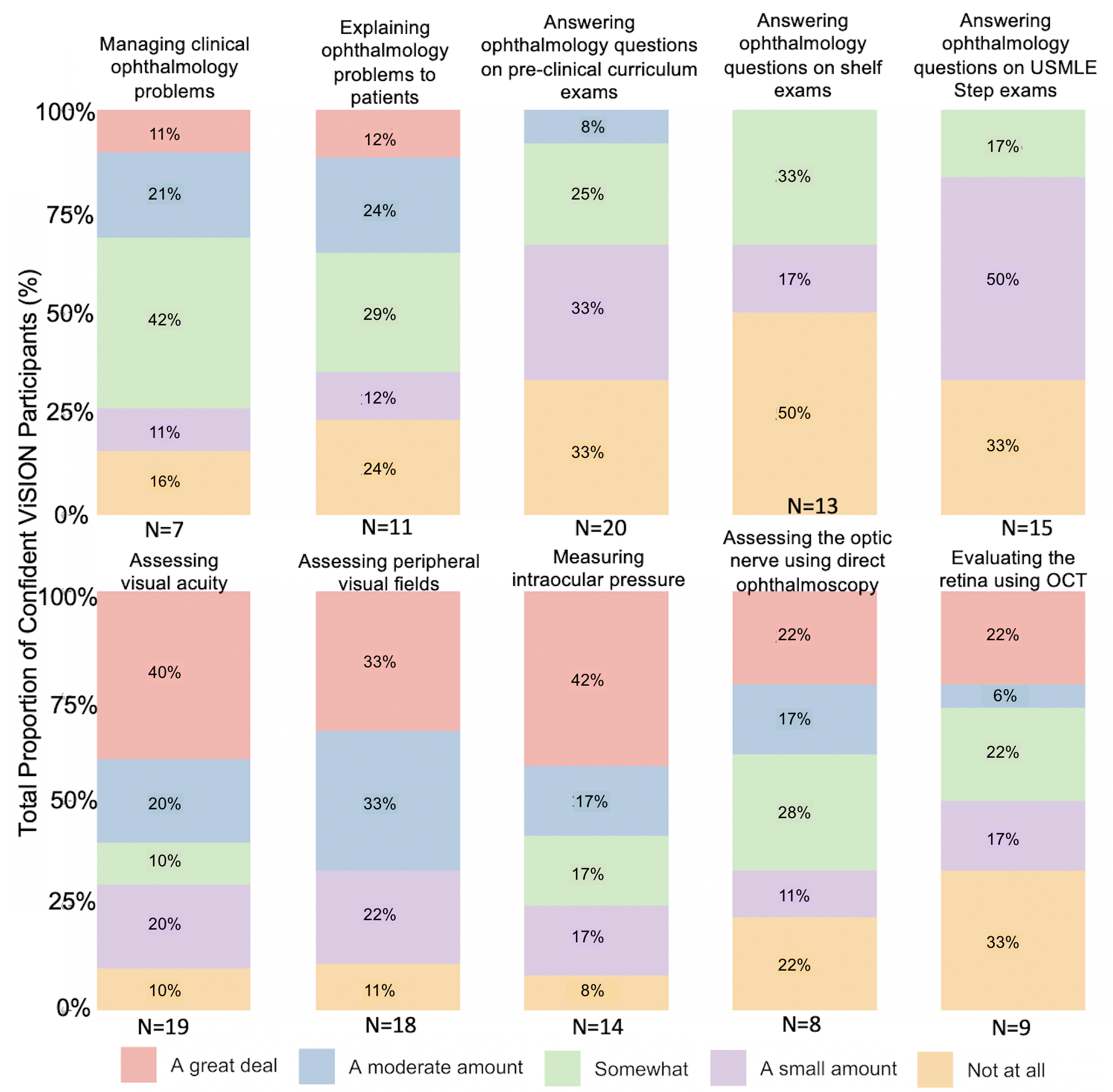

Supplement: Supplementary file 5 — Additional file 5: Figure S2. Survey participant attributions of ophthalmology-related and service-oriented interests, extracurricular participation, and ophthalmology curriculum engagement to participation in ViSION. Stacked bar graphs depict the percentage of respondents that answered each question as indicated in the color-coded legend. Only respondents who indicated interest or participation with a given activity or who indicated feeling at least “somewhat interested or engaged” (level 3 interest on a 5-point scale) in the ophthalmology curriculum were included. [file 12909_2022_3194_MOESM5_ESM.tiff]

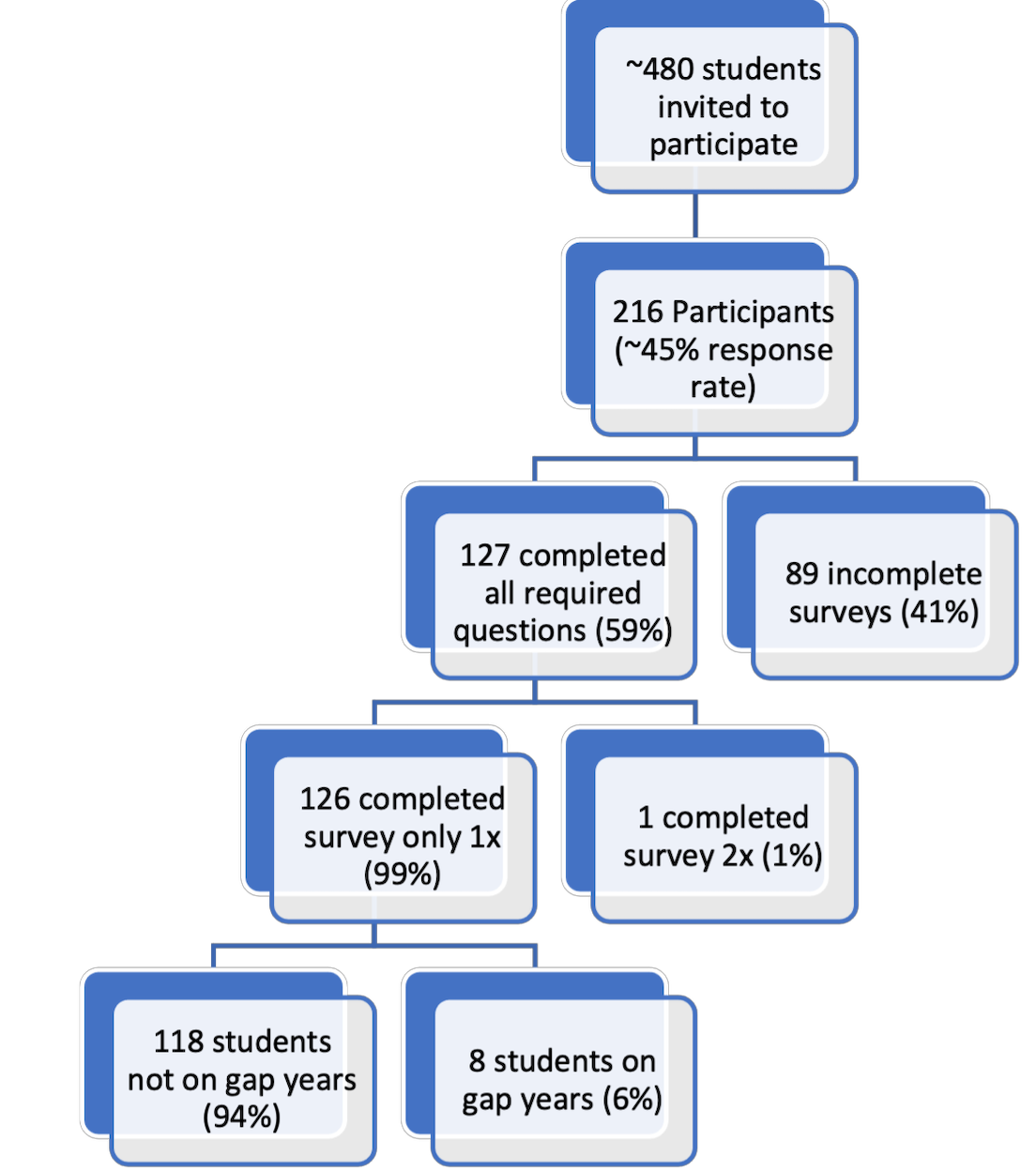

Supplement: Supplementary file 6 — Additional file 6: Figure S3. Survey participant attributions of confidence with ophthalmology clinical skills and exam content to participation in ViSION. Stacked bar graphs depict the percentage of respondents that answered each question as indicated in the color-coded legend. Only respondents who volunteered in the JHU-SOM ViSION Program and indicated feeling at least “somewhat confident” (level 3 confidence on a 5-point scale) performing a given skill or answering exam questions were included. [file 12909_2022_3194_MOESM6_ESM.tiff]
