# Supplementary material for: Demographics, clinical interests, and ophthalmology skills confidence of medical student volunteers and non-volunteers in an extracurricular community vision screening service-learning program
Source: BMC Med Educ. 2022 Mar 4;22:143. doi: 10.1186/s12909-022-03194-0 (PMC8894556; doi:10.1186/s12909-022-03194-0)
Supplement: Supplementary file 7 — Additional file 7: Table S1. ViSION volunteers’ motives for joining, continuing, and terminating ViSION involvement based on survey responses. [file 12909_2022_3194_MOESM7_ESM.docx]

**Supplementary Table S1. ViSION volunteers’ motives for joining, continuing, and terminating ViSION involvement based on survey responses.**

| **Motives for Joining ViSION** |  |
| --- | --- |
| *Interest in Ophthalmology* |  |
| As an MS1, I was not certain whether I wanted to pursue a career in ophthalmology. However, after learning more about the eye/brain in anatomy, and again during NSS (basic science neuro block), I wanted more opportunities to get hands-on experience learning about ophthalmology while also being involved in some kind of outreach/volunteering program. | I am interested in pursuing ophthalmology and I thought it would be a great opportunity to broaden my clinical understanding of ocular diseases and conditions. Equally important, I thought it would be an invaluable chance to learn or pick up skills using ophthalmology equipment that I would otherwise never get the chance to use or practice with. |
|  |  |
| *Community Service* |  |
| I found that this was a group that allowed me to actually learn a skill and volunteer in the community during my preclinical years. There really weren't that many other opportunities through Hopkins that fulfilled those requirements where I felt that I was doing something that was actually making a distinct contribution and both the patients and myself were both advancing. | I wanted to be able to perform service that would actually help patients and link them to care and resources. Additionally, I felt like I could make a difference as visual problems can dramatically alter quality of life. |
| I wanted to help members of the Baltimore community receive free eye screening as this often gets neglected | I wanted to participate in community service focused on working with the local community. |
| Easy, structured, fun way to get community service hours | Help out in the community |
| Joined to help others and strengthen my resume | Looking for clinical community service activities |
|  |  |
| *Both* |  |
| I was interested in pursuing a career in ophthalmology at the time and SSSP seemed like a great way to get involved in the Baltimore community while also learning more about the field. | It seemed like a good volunteer opportunity during which I could learn more about certain exam procedures we don’t generally learn as much about and could at the same time help the community |
| I wanted to learn more about ophthalmology and wanted to serve the Baltimore community |  |
|  |  |
| *Practice Clinical Skills* |  |
| I thought this program would be a great way for me to learn and practice some basic skills in Ophthalmology (IOP, VF, CDR) and think about how to best manage the patients and who to refer for further care. | Since Hopkins Med does not have a specific student-run free clinic, I thought Sight Savers would allow me to do similar things in the ophthalmology context. |
| Wanted to get hands on experience with patients, particularly while working for a cause that has tangible benefits | Practice routine physical exam maneuvers |
| Wanted to feel comfortable using an ophthalmoscope |  |
|  |  |
| *Miscellaneous* |  |
| My friends convinced me to sign up. | Strengthen my resume |
|  |  |
| **Reasons for continuing involvement with ViSION** |  |
| *Community Service* |  |
| I truly do believe that Sight Savers is one of the few programs on campus in which students can have a tremendous impact on our Baltimore community. Training and teaching those younger students to become excited about ophthalmology through vision screening was an absolute joy. Being able to interact with and learn from our own faculty at these screenings was an incredible privilege. Being immersed in our diverse and energized Baltimore community was inspiring and humbling. | One of the main reasons that I continued with SSSP was feeling that I was really able to contribute to bettering the eye health of Baltimore residents. I was especially excited about the program's referral set-up: the ability for patients to get glasses for free or at a discount, for patients to be seen pro bono if they couldn't afford it, and even access to Wilmer funds for surgery if it was necessary, and knowing that I could be a person to help link Baltimore residents to some of these amazing resources motivated me to come to as many screenings as I could. Another reason that kept me coming back to SSSP was getting to work with wonderful faculty. I especially loved working with Dr. Jampel during my first year -- he would spend the entire morning talking both with every volunteer and every patient and getting to know them. |
| Excitement of community members about free glasses and eye care | From volunteering first year, I was so impressed by the organization and excited by the impact we could make as students that I wanted to continue and apply for a board position (which I was selected for). |
| Enjoyed working with children and families in community | I have enjoyed interacting with the Baltimore community |
| I felt that we were doing an excellent job screening patients with eye problems and it was needed in the community | I thoroughly enjoyed working with patients in-need in the Baltimore community, learning more about the social determinants of health, gaining a better understanding of common ophthalmologic diseases |
| My first sight savers event was a great experience. We worked with a majority Spanish speaking population and I speak enough Spanish that I was able to communicate fairly well with the patients. they were so grateful for our help and I could see the difference we made in the community that day by the amount of people we were able to refer for free glasses or for follow up for dangerous eye conditions | Extremely interested in serving Baltimore community while also learning about ophthalmology in a clinical setting |
|  |  |
| *Patient Interaction* |  |
| My first station that I was assigned to was registration and I really appreciated the opportunity to talk to real people. During the school year, chances to interact with patients is rare and so doing activities like this helps me keep centered. I thoroughly enjoy getting the chance to learn on how a different assessment at every new screening. | I really enjoyed the direct patient contact and doing procedures with patients. |
| *Educational Value* |  |
| I enjoyed the vision screenings as an educational resource both about medicine and about Baltimore |  |
| *Miscellaneous* |  |
| Fun working with other students |  |
|  |  |
| **Reason for terminating involvement with ViSION** |  |
| *Time Constraints* |  |
| Clinical rotations became too time consuming and my friends were also no longer going | I have not been very active during clinical year (M3 year) due to rotations but hope to find time to be involved. |
| Clinical rotation time constraints | Had conflicts with scheduled SSSP events |
| I've had a lot of trouble finding free time on the weekends to volunteer with SSSP, especially since I have been on a lot of rotations with weekend shifts lately. I have actually signed up for a number of screenings during my clinical year, but there were so many underclassmen always signed up (usually greater than 15!) that I decided to let them have a chance to learn instead. I'd love to continue coming to screenings as my schedule permits and when extra volunteers are needed! | When you go to the clinical years, your amount of time to dedicate to anything decreases. I unfortunately had to prioritize aspects such as my physical and mental wellbeing while also performing well on rotations. |
| Less time in clinical rotations | I got busy with clinical work |
| Busier with classes and other extracurriculars |  |
|  |  |
| *Lost Interest* |  |
| I took an ophthalmology elective and although I really enjoyed my time with amazing faculty and residents, I could not find a field within Ophthalmology that married my clinical and research interests. In addition, the time constraints of step 1 studying and my PhD work made containing participation in SSSP difficult. | After doing 4-5 events it felt like enough |
